# Revisiting the C60ISO and iso-C60 Data Sets of Relative Energies for C_60_ Isomers

**DOI:** 10.1021/acs.jpclett.6c01636

**Published:** 2026-06-30

**Authors:** Marc Reimann, Martin Kaupp

**Affiliations:** † Universität Innsbruck, Institut für Ionenphysik und Angewandte Physik, Technikerstraße 25/3, A-6020 Innsbruck, Austria; ‡ Technische Universität Berlin, Institut für Chemie, Theoretische Chemie/Quantenchemie, Sekr. C7, Straße des 17. Juni 135, D-10623 Berlin, Germany

## Abstract

We revisit reference data for the widely used relative energies of different isomers of C_60_, previously used in the iso-C60 test set and in the C60ISO subset of the GMTKN55 suite. Using a carefully constructed composite approach, we provide improved isomerization energies of approximate CCSD­(T)-cf/CBS quality. Because of the observed changes of up to more than 10 kcal/mol compared to the previous C60ISO data, we re-evaluated many density functional approximations. While double hybrids improve, long-range-corrected hybrid functionals deteriorate even further. This can be traced back to detrimental effects of large exact-exchange admixture at interelectronic distances just below 2 bohr on the description of intermediate-range correlation effects. While this does not signal multireference-type static-correlation effects, it can be addressed efficiently by modern strong-correlation-corrected range-separated local hybrid functionals. We collected our final combined isomerization energies in the C60iso-17 data set and suggest it should replace the older iso-C60 and C60ISO sets.

## Graphical abstract

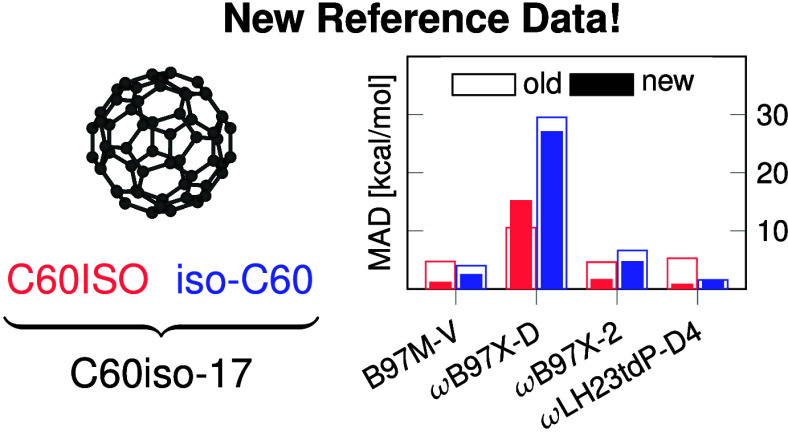

In the pursuit of improved approximate methods in quantum chemistry, in particular of improved approximations to the elusive exchange-correlation functional of Kohn–Sham density functional theory (DFT), high-quality reference data sets have been crucial for validation and training. A particularly widely used benchmark is the GMTKN55 suite of main-group thermochemistry, kinetics, and noncovalent interactions.^1^ The subset with the overall largest molecular sizes within GMTKN55 is the C60ISO set,^2^ which contains 10 isomers of C_60_ and their relative energies obtained at the DLPNO-CCSD­(T)/CBS* level. Already in the original work,^1^ the authors noticed that double hybrid functionals, i.e., the rung 5 set of functionals providing the overall best performance for the entire GMTKN55 suite, gave larger deviations from the reference data than simple hybrid functionals. This led to the conclusion that these C_60_ systems were more challenging than many other subsets.^1^ Shortly after the publication of both C60ISO and GMTKN55, the iso-C60 data set was published,^3^ using high-level G4­(MP2) reference energies for a different set of eight C_60_ isomers out of the same superset sampled in ref 2, but referring to the same lowest-energy structure of *I*_*h*_ symmetry. The same level of theory has since been used to study a set of C_40_ isomers.^4,5^ Comparison of the results of the two studies shows surprising differences. At the PBE-D3 level, ref 2 found a mean absolute deviation (MAD) of 10.8 kcal/mol in the C60ISO set while ref 3 found an MAD of only 6.3 kcal/mol for the iso-C60 set. With global hybrid PW6B95-D3, the deviation is even more pronounced, with MADs being only 1.6 kcal/mol for the C60ISO set and a staggering 12.9 kcal/mol for the iso-C60 data set. While the latter does include different and overall higher-lying isomers, these differences are much larger than expected. A lack of systematic comparability of the performance of different functionals for the two sets had also been noted elsewhere.^6^ We assumed that these large differences should likely be traced back to the different methods used to obtain the reference energies. We note in passing that the ordinal numbers of two isomers in the C60ISO main text (**196** and **266**) do not correspond to the numbering in the Supporting Information of the same paper and should have been **195** and **265**, respectively. Reference 3 investigated the energies of isomers **6**, **8**, **265**, **303**, **576**, **795**, and **1748** relative to isomer **1**.^7^ Both studies therefore include an identical isomerization reaction from global minimum **1** to the *C*_2*h*_ symmetric structure with ordinal number **265**. The isomerization energies, however, differ by 10.5 kcal/mol, clearly showing a large discrepancy between both sets of energies. We have been motivated to revisit these data due to our experience in using GMTKN55 and C60ISO for the evaluation of our own new local hybrid functionals, where some of the comparisons seemed nonintuitive.^8^ Due to significant advances in local coupled cluster (CC) methods that can treat larger and larger systems accurately, we revisit here the benchmark data of the C60ISO and iso-C60 data sets. This also allows us to re-evaluate the performance of different density functionals against the improved reference data, giving an updated view of some previous interpretations. We note that the relative energies of fullerene isomers are of wide interest in their own right regarding formation and derivatization reactions, as well as a general understanding of the relations between structure and stability in the contexts of the curvature of large π systems, spin frustration, strain, static and dynamic electron correlation, and the isolated pentagon rule (see, e.g., refs 9–11 ).

We base our reference energies on the PNO-LCCSD­(T)-F12b approach of Ma and Werner^12,13^ that allows for an efficient energy evaluation even for large systems. Even with this approach, however, the basis set had to be restricted to a modified cc-pVDZ-F12 basis (see ) to reduce the computational burden. Using an incremental approach, we introduce corrections for core correlation, basis set incompleteness, and the local PNO domains. We additionally added estimates for “beyond-CCSD(T)” higher-order excitation contributions using the continued fraction (cf) approach.^14^ The final best estimates are obtained from eq 1, while the individual contributions are explained in detail in the .

1 $$E_{\mathrm{final}}=E_{\mathrm{PNO}‐\mathrm{LCCSD}\left(T*\right)‐F12b}^{{\mathrm{VDZ}‐F12}^{\prime }}+\delta E^{\mathrm{core}}+\delta E^{\mathrm{basis}}+\delta E^{\mathrm{PNO}}+\delta E^{\mathrm{post}‐\mathrm{CCSD}\left(T\right)\mathrm{,cf}}$$

We note in passing that all of our calculations are based on restricted HF wave functions, even though these do not produce the lowest energies at the HF level.^9^

As we perform our calculations on both sets of isomers, our results give rise to a new benchmark set of 17 isomerization energies of C_60_ isomers that we label as C60iso-17. Our new best estimates of isomer energies (relative to isomer **1** with *I*_*h*_ symmetry) are compared to the original data for C60ISO and iso-C60 in Table 1.

**Table 1:** Relative Isomer Energies Obtained in This Work and Earlier Works (all values in kilocalories per mole)

isomer^a^	C60ISO^b^	iso-C60^c^	C60iso-17, this work^d^
**1**	0.00	0.00	0.00
**2**	37.40	–	37.39
**3**	56.87	–	55.93
**4**	56.89	–	55.79
**5**	69.89	–	68.78
**6**	–	73.49	74.60
**8**	–	74.43	75.17
**20**	100.48	–	92.32
**42**	111.72	–	106.46
**195**	142.18	–	130.64
**196**	–	–	131.48
**265**	143.96	133.46	136.12
**266**	–	–	136.83
**303**	–	134.44	136.42
**576**	–	155.00	157.95
**580**	164.88	–	158.39
**795**	–	165.44	170.11
**1748**	–	256.69	260.03

a Isomer numbers from ref 2, corrected as indicated in the text.

b From ref 2.

c From ref 3.

d According to eq 1.

The contributions of the different corrections to the PNO-LCCSD­(T*)-F12b base values are reported in . Since the core-correlation corrections (*δE* ^core^) are all less than 0.8 kcal/mol, we expect that MP2 is sufficient to give a reliable estimate and that the remainder of the calculations can be performed with a frozen core. Basis set corrections (*δE* ^basis^) are generally less than 0.4 kcal/mol, indicating that the chosen F12 treatment with a modified cc-pVDZ-F12 basis practically converges to the CBS limit. To account for residual errors in the PNO approach (*δE* ^PNO^), we perform a two-step correction, where we test the effect of tightened PNO thresholds at the noniterative PNO-LCCSD­(T*_0_)-F12b level and combine the result with the remaining PNO error at the (PNO-L)­MP2-F12 level (see the for further details). These corrections are almost negligible for the lower-lying isomers (**1–8**) but increase to a few kilocalories per mole for the higher-lying ones. Isomer **1748** exhibits a major contribution of more than 14 kcal/mol, indicating a more challenging PNO-domain construction for this isomer. An alternative, one-step estimation at the (PNO-L)­MP2-F12 level provides very similar results ().

Using just these three contributions, we expect our results to be close to the canonical CCSD­(T)/CBS quality. We should, however, discuss whether single-reference CCSD­(T) is even a suitable benchmark level for these isomerization energies. Recent careful computational analyses of fullerene isomerization energies and of correlation contributions to them^10^ concluded that C_60_ (in its *I*_*h*_ symmetric minimum) is not a strong-correlation case. This is significant, as earlier work showed pronounced spin-symmetry breaking at the restricted Hartree–Fock level even for the lowest-lying C_60_ isomer.^9^ However, Lee and Head-Gordon^10^ concluded that this instability is artificial rather than indicative of strong correlations, in contrast to smaller fullerenes like C_20_ and C_36_. Rather than exhibiting local strong correlations, the static correlation in C_60_ consists of an accumulation of more moderate medium-range (see further below) correlations around the many carbon atoms.^10^ These observations would suggest that CCSD­(T) is a suitable benchmark method. To obtain further insight, we initially investigated standard *T*_1_ and *D*_1_ coupled cluster diagnostics (). We observed rather similar values for most isomers, with a slight increase in the *D*_1_ value to more than 0.065 for isomers **195** and **795**. This is slightly above the typically used multireference threshold of 0.05 but below the threshold proposed for transition metal compounds, for which multireference problems occur much more often.^15^ Neither diagnostic clearly suggests the presence of strong correlation.

Therefore, we expect that an approximate estimate of higher-order coupled cluster excitations via the continued fraction method^14^ (*δE* ^post‑CCSD(T),cf^ (see )) is valid. These contributions are only around 1 kcal/mol up to isomer **8** and then slowly increase to almost 7 kcal/mol for isomer **1748**. This indicates an increase in correlation strength for the higher-lying isomers, in agreement with expectation. However, these contributions are less than 3% and thus are largely consistent with the C_60_ isomers not being strong-correlation cases^10^ (we note that in spite of the spin-symmetry breaking at the HF level, even in ref 9 the authors acknowledged that single-reference methods should be adequate for computations on C_60_).

To further investigate the correlation effects in fullerenes, we computed fractional occupation densities (FODs)^16^ at the BHLYP/def2-TZVP level (at 15 000 K). These analyses found relatively large *N* ^FOD^ values between 5.1 and 7.8 for the different isomers. This value is the spatial integral of the corresponding fractional occupation density (ρ^FOD^) and was found to correlate with multireference character for various compounds.^16,17^ The obtained values are relatively high and would clearly signal multireference character in smaller molecules.^17^ However, visual inspection showed the presence of ρ^FOD^ amplitudes in all carbon 2p orbitals orthogonal to the surface of the fullerenes, pointing outside the cage (see for isomer **1**). Since the number of contributing atoms is therefore rather high, we suggest the use of the mean atomic contribution to *N* ^FOD^ (which we denote as $n_{\mathrm{mean}}^{\mathrm{FOD}}$ ) and the maximum/minimum atomic contribution (denoted as $n_{\max}^{\mathrm{FOD}}$ / $n_{\min}^{\mathrm{FOD}}$ ). The obtained results are listed in . For the sake of simplicity, we restrict the analysis to the FOD contributions created by p orbitals. All atomic contributions are shown in the . The analysis suggests that the increase in *N* ^FOD^ for all isomers above **1** can be largely traced back to an increase in the atomic contribution by a significant number of carbons but not by all of them.

We further address the suitability of our CCSD­(T)-cf-based protocol by investigating a simple model system, for which we can perform explicit CCSDT­(Q) calculations (see the for details). We model the different vertices of C_60_ isomers by a stepwise bending of the C–H bond in benzene from the plane to a maximum φ angle of 90°, retaining *C*_*s*_ symmetry (see Figure 1). This breaks the conjugation of the system by tilting the corresponding p orbital of the carbon atom out of the plane. The $n_{\max}^{\mathrm{FOD}}$ values that can be observed in such a treatment are indeed very close to those observed in the C_60_ isomers for similar tilting angles (see ). We think this makes this model better suitable than previous ones like, e.g., those in ref 18.

**Figure 1:**
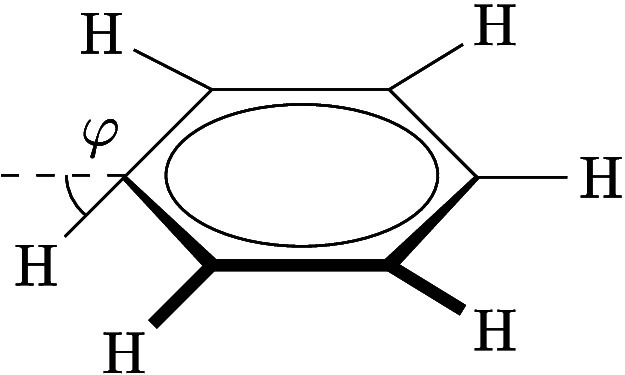
Structure of the benzene-based model system used in this study. The system has *C*_*s*_ symmetry, and φ is varied between 0° and 90°.

The model system clearly shows an increasing deviation of the relative CCSD­(T)-cf energies from reference values with an increase in φ (see ). However, the deviation is only on the order of 0.1 kcal/mol. If we assume a functional dependence between the deviation and the $n_{\max}^{\mathrm{FOD}}$ values that is asymptotically linear (see ), we can estimate additional “post-CCSD­(T)” contributions for the fullerene models by adding the corresponding contribution for every carbon atom in a fullerene. While this could be added to the reference energies (), we prefer to use the effect to estimate an uncertainty for our reference energies based on eq 1. Adding this correction changes the energies by up to 4 kcal/mol or up to 2% (for isomer **1748**, see ). Notably, uncertainties for the lower-lying isomers are much smaller. Replacing the cf correction plus the smaller on-top model corrections () entirely by a “post-CCSD­(T)” correction based on the benzene model system () gives results within 1.3 kcal/mol, indicating the mutual consistency of the two approaches.

Based on these considerations, we think that the best estimates in Table 1 based on eq 1, which include the beyond-CCSD­(T) continued fraction contributions, are suitable benchmark data that supersede the values from the two previous works. Additional contributions based on our distorted benzene model depend on the significant assumption of their linear dependence on $n_{\max}^{\mathrm{FOD}}$ values. We therefore refrain from including them in the CCSD­(T)-cf-based best estimates but find them useful to provide an uncertainty measure of the latter, which appears to be maximally 2% for the highest-lying isomers. The employed best estimates based on eq 1 are lower than the original C60ISO reference values, only marginally so for the lowest-lying isomers, but increasingly for the higher-lying ones, up to 11.5 kcal/mol for isomer **195** and 7 kcal/mol for isomer **580**. In contrast, our best estimates are closer to the original iso-C60 values but are in this case somewhat larger, by up to 4 kcal/mol for isomer **795**. The original C60ISO reference data^2^ were also obtained with local coupled cluster [DLPNO-CCSD­(T)] calculations, using a still relatively early version of the code. Thus, they had to employ semicanonical treatments of the perturbative triples corrections, typically labeled (*T*_0_), as the full iterative procedure was implemented only later.^19^ In our calculations, we find a difference between (*T*_0_) and full (*T*) corrections of up to 1.5 kcal/mol for the original C60ISO test set. Also, the default PNO cutoff value of 3.33 × 10^–7^ might lead to significant residual errors. Although our PNO-based results cannot be directly compared to the DLPNO framework, we see changes of up to 1 kcal/mol when we tighten the PNO thresholds for the same set. We can, however, trace back the most significant part of the differences to their semiempirical corrections to the complete basis set limit (termed MP2/CBS*), which deviate from our MP2/CBS and MP2-F12 results by up to 6.5 kcal/mol (see ). The original reference level for iso-C60 is the G4­(MP2) method, which augments canonical CCSD­(T) calculations in the rather small 6-31­(d) basis set by relatively large basis set corrections at the MP2 level. The relatively good agreement between these data and our new results might be in part due to error compensation. Our new reference energies include very carefully constructed corrections for all major remaining sources of errors of a PNO-LCCSD­(T*)-F12b/VDZ-F12′ treatment, indicating relatively small corrections for core correlation or basis-set incompleteness, and they even estimate most effects beyond CCSD­(T). They should therefore be considered more accurate than the older results.

We note in passing that we have also performed reference energy calculations with the same new composite approach for the UPU23 test set of RNA dinucleotide backbone structures,^20^ which is also part of the GMTKN55 suite and had previously been referenced at the same DLPNO-CCSD­(T)/CBS* composite level as C60ISO (see ). While the percentage deviations are sizable for several structures, the unsigned relative energies change by at most 1 kcal/mol. This suggests that the approximations made in the previous work are less severe for UPU23 than those for C60ISO. However, since the overall energy differences of UPU23 are smaller than those of C60ISO, the (relatively small) maximum impact of the new reference data on the overall WTMAD-2 values of GMTKN55 would be about 3 times larger than the maximum expected change for the C60ISO test set (0.06 and 0.02 kcal/mol, respectively).

Given the differences specifically with respect to the original C60ISO data, with relative energies reduced by up to 11.5 kcal/mol (isomer **195**), we will re-evaluate various density functional approximations (DFAs) using the newly generated, more accurate reference energies listed in Table 1. Detailed statistical data for a wider range of DFAs against the new data for the isomers from the C60ISO, iso-C60, and combined C60iso-17 sets are provided in , while comparisons to the older reference data can be found in . We will not discuss in detail the effects of dispersion corrections, as they influence performance relatively little (and we use mostly D4 corrections, while many previous studies used D3(0) or D3­(BJ) terms).

Figure 2 shows the mean absolute deviations (MADs) for the most widely used C60ISO set with both the old and the revised reference values for a selection of DFAs. As our new reference isomerization energies are generally lower than the original values, most notably for the higher-lying isomers, we expect the performance of those DFAs that systematically underestimated the original values to show improved performance and those DFAs that overestimated to perform even worse. This is borne out by the numerical data in . GGAs, which had mean signed deviations (MSDs) around −11 kcal/mol, are improved to a range around −6 kcal/mol. meta-GGA functionals like TPSS, or MN15-L, which already had smaller negative MSDs, perform even better now. The meta-GGA-based composite method r^2^SCAN-3c also performs exceptionally well, with an MAD close to 1 kcal/mol. Among global hybrid functionals with low exact-exchange (EXX) admixtures, TPSSh and O3LYP stand out. Originally, they had an MSD around −5 kcal/mol; now they perform outstandingly, with MADs below 1 kcal/mol. Global hybrids with larger EXX admixtures like M06-2X, which already overestimated the energy differences, perform even worse now. The originally top-performing PW6B95, PBE0, and TPSS0 functionals are now more clearly overestimating the reference data. Local hybrid (LH) functionals like LH20t^21^ perform like GHs with moderate EXX admixtures. Interestingly, some strong-correlation-corrected variants like scLH22t^22^ improve performance significantly by shifting the energy differences to lower values.

**Figure 2:**
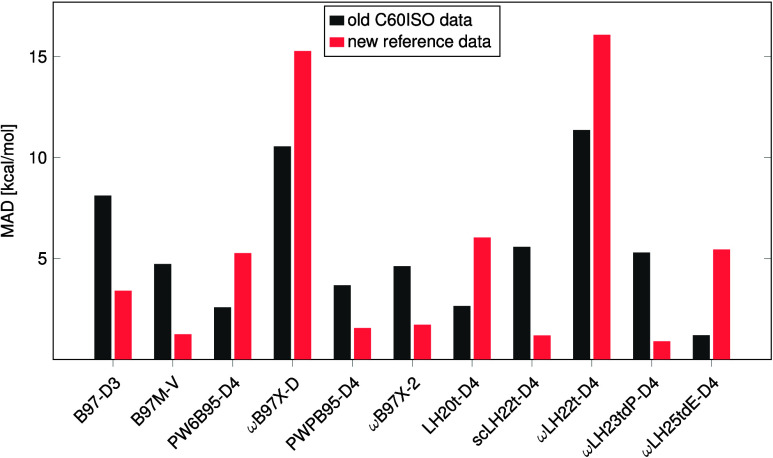
MADs for selected DFAs in kilocalories per mole for the C60ISO set of C_60_ isomerization energies against the new and older reference energies provided in Table 1 (cf. ).

It had already been noted that range-separated hybrid (RSH) functionals with large long-range EXX admixtures like the ωB97 series overestimate the C60ISO data by ≥10 kcal/mol. With the revised reference data, they perform even worse ( and Figure 2); e.g., ωB97X-D or ωB97M-V exhibits MADs of 15–16 kcal/mol. These results and the results obtained by varying the parameters of the functional (see ) confirm that such long-range corrected hybrids exhibit large deviations for C60ISO (as well as for iso-C60), even more so now with the new reference data. This appreciable overestimate also extends to the ωLH22t range-separated local hybrid (RSLH ()).^23^ While this functional has position-dependent EXX admixture at short range, like the ωB97 functionals its admixture increases toward 100% at long range, which is obviously not helpful in the present context.

This makes the observation particularly interesting that strong-correlation-corrected variants of ωLH22t, such as ωLH23tdX (X = B, E, or P) and ωLH25tdE, improve dramatically upon the performance of their parent RSLH (). ωLH23tdP performs particularly well (Figure 2). The construction of these functionals (see ) includes a coordinate-space detection function that spots strong correlations.^24–26^ When this occurs, the local mixing function (LMF) governing mostly the short- to intermediate-range EXX admixture is reduced locally by the strong-correlation function *q*_AC_(**r**) (; this aspect holds already for strong-correlation-corrected LHs like scLH22t; see above). Additionally, in the same spatial regions, the full long-range EXX admixture of ωLH22t is sacrificed in part by an additional term governed by switching function *f*_FR_(**r**) () that depends on the same coordinate-space detection function as *q*_AC_(**r**).^24–26^ Closer analysis (see for the performance of modifications of ωLH23tdE) indicates that both aspects of sc corrections in RSLHs are important for performance on C60ISO, with inclusion of *q*_AC_(**r**) in the LMF giving a somewhat larger improvement.

The coordinate-space functions that govern these modifications by both *q*_AC_(**r**) and *f*_FR_(**r**) exhibit large amplitudes in the same radial direction outside the cage as discussed above for ρ^FOD^. While these terms have been labeled “strong-correlation corrections”,^22,24–26^ we may view them in the context discussed above, where smaller correlations on many carbon atoms add up to make a significant contribution to electronic structure and relative energies. In other words, while static correlation is detected and corrected, this does not amount to a multireference situation. We find this a notable additional indication that these strong-correlation-corrected RSLHs exhibit a significant escape from the usual zero-sum game between minimizing delocalization and static-correlation errors.^26,27^ For example, full long-range EXX admixture in RSH or RSLH functionals is crucial for obtaining accurate quasiparticle energies in generalized Kohn–Sham calculations^28^ (e.g., to compute ionization potentials, electron affinities, or band gaps using Koopmans’ theorem), to get reliable charge-transfer excitations within a TDDFT framework,^23^ or to improve on cases in which delocalization errors are crucial for reaction energies, e.g., in a recent “thermometer for nonlocal exchange”.^29^ At the same time, such long-range EXX admixture is detrimental for situations with significant static correlation, such as the spin-restricted dissociation of covalent bonds.^24^ We may add the relative energies of C_60_ isomers to this category. Even if the type of correlation effects found here are not easily classifiable, they are clearly picked up by the correction terms introduced into these functionals. It is most notable that scRSLHs like the ωLH23tdX series or ωLH25tdE generally are found to improve on these C_60_ energetics or on spin-restricted bond dissociation^24–26^ without sacrificing performance for the cases mentioned above in which delocalization errors can be crucial.

We finally look at double hybrid (DH) functionals. In the original evaluations, these underestimated the C60ISO isomerization energies by about 4–7 kcal/mol. Consequently, with the new reference data, DHs are now among the best-performing functionals, with MADs between 1 and 3 kcal/mol (). Unlike for the global and local hybrids, introduction of range separation does not systematically deteriorate results, or at least less so. Only ωB2PLYP, which interpolates between 53% short-range and 100% long-range EXX admixture using an ω of 0.30, shows a very large MAD. We have studied further versions of this ωB2PLYP-type DH with modifications of the EXX admixture as a function of interelectronic distance (see ). Clearly, the main performance differences are related to the region below 2 bohr. Comparable EXX admixture in this regime leads to comparable MADs, independent of the admixture at longer distances. Adding more EXX in this regime increases the MAD significantly. Bonding in C_60_ is characterized by a situation in which Hartree–Fock overlocalizes the C–C bonds in the C_60_ cages.^9,10^ It seems likely that the overly large EXX admixture in this interelectronic distance range leads to an underestimate of intermediate-range correlation effects, which also manifest in the FOD and the coordinate-space quantities of strong-correlation corrections in LHs (see above).

We briefly discuss also the iso-C60 performance of DFAs. Here the shift of the reference data is overall smaller and in the opposite direction, i.e., toward overall somewhat higher values. As iso-C60 contains more higher-lying isomers, the isomerization energies tend to be overall larger, and the statistical deviations of DFAs also tend to be more pronounced. GGA functionals provided underestimates by ca. 6 kcal/mol in ref 3. This increases now to about 9 kcal/mol. meta-GGA functionals that originally had negative MSDs perform somewhat worse now, while those that had positive MSDs perform better (). The meta-GGA-based composite method r^2^SCAN-3c again performs excellently. We can transfer the arguments made above on global hybrids with small or large EXX admixtures analogously to iso-C60, noting that deviations tend to be overall larger for the latter functionals. The same holds for the large overestimate by RSH and RSLH functionals with large long-range EXX admixtures and for the dramatic improvement over ωLH22t by its strong-correlation-corrected variants. Double hybrids had already somewhat negative MSDs for iso-C60 originally^3^ and are shifted to somewhat more negative ones with the new reference data (). In spite of the overall good perfomance of DHs, we confirm the conclusions of refs 3 and 6 that they do not perform noticeably better than a number of simpler functionals from the lower rungs, and that therefore the usual assumption of a systematic improvement when climbing Jacob’s ladder of DFAs^30^ is not strictly fulfilled for these particular isomerization energies.

In conclusion, new high-quality reference data for the isomerization energies of a set of 18 isomers of C_60_ have been generated based on local CCSD­(T)-F12 calculations. This new C60iso-17 set spans two previous ones, namely, C60ISO and iso-C60. While our new reference energies are only moderately larger than the literature values of the iso-C60 set,^3^ they are appreciably lower than the original C60ISO reference data,^2^ up to 11.5 kcal/mol for some higher-lying isomers. The reliability and accuracy of the present reference data have been evaluated in detail and are expected to be better than the previous benchmark data, likely with an error margin of less than 2%. Unlike previous values, which aimed to reproduce only CCSD­(T) quality, we also include estimates for higher-order excitations. While some beyond-CCSD­(T) contributions are noted, the C_60_ isomers are not true strong-correlation cases, in agreement with recent analyses.^10^ This is shown also by our evaluations of approximate density functionals and the fact that double hybrid functionals with their large exact-exchange admixture and MP2 correlation contributions in fact perform quite well here, better than assumed from the earlier C60ISO evaluations. In contrast, for the latter subset, the poor performance of range-separated hybrid functionals with large long-range exact-exchange contributions is even worse with the new reference data and is attributed to an insufficient description of cumulative intermediate-range correlation contributions that concentrate radially on the surface of the C_60_ cages. Interestingly, these contributions are captured reliably by a number of coordinate-space “strong-correlation corrections” added to local hybrid and range-separated local hybrid functionals, therefore improving performance significantly. We recommend the presented combined C60iso-17 set, or its two original subsets, with the new reference values as a sensitive test of approximate quantum-chemical methods. Use in combination with test sets, where deviations with various DFAs tend to be dominated by delocalization errors, seems to be particularly informative regarding the frequent zero-sum trade-offs observed between modeling certain types of electron correlation and minimizing the consequences of self-interaction errors by including Hartree–Fock-like exchange.

## Supplementary Material





## Notes

Electronic energies and detailed results of FOD analysis (XLSX)
Computational details, additional results, and additional discussion of range-separated local hybrids (PDF)
