# Supplementary material for: Revisiting the C60ISO and iso-C60 Data Sets of Relative Energies for C60 Isomers
Source: J Phys Chem Lett. 2026 Jun 30;17(28):8077–82. doi: 10.1021/acs.jpclett.6c01636 (PMC13383827; doi:10.1021/acs.jpclett.6c01636)
Supplement: Supplementary file 2 [file jz6c01636_si_002.pdf]

Supporting Information: Revisiting the C60ISO and iso-C60 datasets of relative energies for C_{60} isomers

Marc Reimann*,† and Martin Kaupp*,‡

†*Universität Innsbruck, Institut für Ionenphysik und Angewandte Physik, Technikerstr. 25/3, A-6020, Innsbruck, Austria*

‡*Technische Universität Berlin, Institut für Chemie, Theoretische Chemie/Quantenchemie, Sekr. C7, Straße des 17. Juni 135, D-10623, Berlin, Germany*

E-mail: marc.reimann@uibk.ac.at; martin.kaupp@tu-berlin.de

Computational Details

All calculations were performed on structures taken from Ref. S1. All explicitly correlated, PNO-based calculations were performed with the MOLPRO program, version 2023.2,[S2,S3] using the implementation of Ma and Werner.[S4–S6] All calculations employed the cc-pVDZ-F12 basis set[S7] and the appropriate auxiliary basis set loaded automatically by the program. Due to small eigenvalues of the overlap matrix, the most diffuse set of p-functions of the cc-pVDZ-F12 basis set was removed in all calculations (denoted as VDZ-F12'). Calculations employed either default options for the PNO calculations or the DOMOPT=tight option for a numerically improved domain construction. All energies include the CABS singles correction.[S8] Unless noted otherwise, all calculations employed a frozen core, which excluded the 1s orbitals of carbon from the correlation treatment. PNO coupled cluster calculations employed the CCSD-F12b formalism.[S8] The effect of the explicit correlation to the perturbative triples contributions was estimated using scaled (T) contributions with either the constant scaling factor proposed by Peterson et al.[S9] (termed (T_s)) or a scaling factor obtained by the ratio of explicitly correlated and standard MP2 energies proposed before[S10,S11] (termed (T*)). Unless explicitly noted otherwise, all perturbative triples contributions were obtained with the proper iterative procedure[S6,S12] and not with the semi-canonical approach (T_0). To estimate effects of higher order excitations, we employ the continued fraction (cf) approach of Goodson[S13]

$$E_{\text{CCSD(T)-cf}} = \frac{\delta_1}{1 - \frac{\delta_2/\delta_1}{1-\delta_3/\delta_2}} \tag{S1}$$

with:

$$\delta_1 = E_{\text{SCF}} \tag{S2}$$

$$\delta_2 = E_{\text{CCSD}} - E_{\text{SCF}} \tag{S3}$$

$$\delta_3 = E_{\text{CCSD(T)}} - E_{\text{CCSD}}. \tag{S4}$$

$$\tag{S5}$$

Canonical MP2 and MP2-F12 calculations were performed using the ORCA program, Version 5.0.2.[S14] MP2-F12 calculations employed the same settings as the PNO-LMP2 calculations described above, unless explicitly stated otherwise. Canonical MP2 calculations employed cc-pV(T,Q,5)Z basis sets[S15] and the corresponding auxiliary basis sets for the resolution of the identity (RI).[S16]

Hartree-Fock calculations were in all cases performed by similar density fitting approaches (RIJK) using the appropriate auxiliary basis sets.[S17] All explicitly correlated methods employed an exponent $\beta = 1.0$ for the Slater-type frozen geminal.

The final energies were obtained at PNO-LCCSD(T*)-F12b/VDZ-F12' level, augmented by corrections for core correlation, basis-set incompleteness, the incompleteness of the PNO space, and the continued-fraction estimate for higher-order excitations:

$$E_{\text{final}} = E^{\text{VDZ-F12'}}_{\text{PNO-LCCSD(T*)-F12b}} + \delta E^{\text{core}} + \delta E^{\text{basis}} + \delta E^{\text{PNO}} + \delta E^{\text{post-CCSD(T),cf}} \tag{S6}$$

This equation is identical to eq. 1 in the main text. The first two correction terms are evaluated at the MP2 level. The first one corrects for core correlation by taking the difference between an all-electron (AE) and a frozen-core (FC) MP2 calculation

$$\delta E^{\text{core}} = E^{\text{CVTZ}}_{\text{AE-MP2}} - E^{\text{CVTZ}}_{\text{FC-MP2}}, \tag{S7}$$

while the second corrects towards the complete basis set (CBS) limit at the MP2 level as

$$\delta E^{\text{basis}} = E^{\text{CBS(T,Q,5)}}_{\text{MP2}} - E^{\text{VDZ-F12'}}_{\text{MP2-F12}}, \tag{S8}$$

using separate extrapolation schemes for the SCF and the correlation energy and employing data obtained with cc-pVXZ (X=T,Q,5) basis sets:

$$E^{\text{CBS(T,Q,5)}}_{\text{MP2}} = E^{\text{CBS(T,Q,5)}}_{\text{HF}} + E^{\text{CBS(T,Q,5)}}_{\text{corr., MP2}} \tag{S9}$$

The Hartree-Fock energies were extrapolated using the approach proposed by Feller [S18] as

$$E^{X}_{\text{HF}} = E^{\text{CBS}}_{\text{HF}} + A\exp(-C \cdot X) \tag{S10}$$

whereas correlation energies were extrapolated using

$$E^{X}_{\text{corr., MP2}} = E^{\text{CBS}}_{\text{corr., MP2}} + B \cdot (X - p)^{-3} \tag{S11}$$

The PNO error was evaluated in a stepwise procedure, using the PNO-LCCSD(T_0)-F12b and the (PNO-L)MP2-F12 level:

$$\delta E^{\text{PNO}} = \delta E^{\text{PNO}}_{(1)} + \delta E^{\text{PNO}}_{(2)} \tag{S12}$$

$$\delta E^{\text{PNO}}_{(1)} = E^{\text{VDZ-F12'}}_{\text{PNO-LCCSD(T}_0\text{*)-F12b,domopt=tight}} - E^{\text{VDZ-F12'}}_{\text{PNO-LCCSD(T}_0\text{*)-F12b,domopt=default}} \tag{S13}$$

$$\delta E^{\text{PNO}}_{(2)} = E^{\text{VDZ-F12'}}_{\text{MP2-F12}} - E^{\text{VDZ-F12'}}_{\text{PNO-LMP2-F12,domopt=tight}} \tag{S14}$$

The second term ($\delta E^{\text{PNO}}_{(2)}$) tries to capture the residual error of the PNO approximation using

tight thresholds compared to canonical calculations. The other term ($\delta E^{\text{PNO}}_{(1)}$) estimates the error introduced by the necessary loosening of the thresholds for the full PNO-LCCSD(T) calculations. Alternatively, the PNO error could be calculated in a single step at the MP2 level:

$$\delta E^{\text{PNO}}_{\text{one-step}} = E^{\text{VDZ-F12'}}_{\text{MP2-F12}} - E^{\text{VDZ-F12'}}_{\text{PNO-LMP2-F12,domopt=default}} \tag{S15}$$

The contributions of higher-order excitations were estimated as

$$\delta E^{\text{post-CCSD(T),cf}} = E^{\text{VDZ-F12'}}_{\text{PNO-LCCSD(T*)-F12b-cf}} - E^{\text{VDZ-F12'}}_{\text{PNO-LCCSD(T*)-F12b}} \tag{S16}$$

following eq. S1. The results are shown in Tables S1 and S4.

We have performed MP2-F12 calculations with different values for β (see Table S5), with the (T_s) correction (using the constant scaling factor of 1.1413 proposed by Peterson et al.[S9]), with the one-step PNO correction, and without the cf approach for comparison (see Table S4), but the effects are small in all cases.

Additional calculations for the benzene-based model system were performed using MRCC,[S19,S20] (release date May 23, 2018) using aug-cc-pVDZ basis sets on the CH unit of interest and the neighboring C atoms and cc-pVDZ basis sets on all other atoms. FCI energies were approximated using a Padé approximant analogous to the one proposed by Goodson[S13]

$$E_{\text{CCSDT(Q)-r}} = \frac{\delta_1(\delta_4(\delta_2+\delta_3) - \delta_3^2 - \delta_4^2) + (\delta_2^2 - \delta_4 - \delta_2\delta_3(\delta_3 - 2\delta_4) - \delta_4^3)}{\delta_4(\delta_2+\delta_3) - \delta_3^2 - \delta_4^2}, \tag{S17}$$

with

$$\delta_3 = E_{\text{CCSDT}} - E_{\text{CCSD}} \tag{S18}$$

$$\delta_4 = E_{\text{CCSDT(Q)}} - E_{\text{CCSDT}}. \tag{S19}$$

$$\tag{S20}$$

Most density functional calculations were performed using a locally modified version of the TURBOMOLE program package.[S21,S22] All calculations employed def2-QZVPP basis sets,[S23] the MARIJ approximation[S24] and a suitable auxiliary basis set as well as a fine integration grid (gridsize 5). EXX computation for global and range-separated hybrid functionals was sped up using semi-numerical integration[S25] with the smaller gridsize 3 and de-aliasing (do_sfit). In contrast, for local hybrids and their range-separated extensions, the full grid (gridsize 5) is used also for the EXX energy density. All calculations were converged to energy changes below 10^{-9} Hartree. Whenever applicable, calculations included the D4 dispersion correction.[S26] We performed calculations using: The GGA functionals BLYP,[S27–S29] BP86[S27,S30,S31] and PBE;[S32,S33] the meta-GGA functionals B97-D3[S34,S35] and TPSS[S36] as well as the composite approach r^{2}SCAN-3c;[S37] the global hybrid functionals TPSSh,[S38,S39] B3LYP,[S40] O3LYP,[S41] PBE0,[S42] TPSS0,[S43] PW6B95,[S44] BMK,[S45] BHLYP,[S46] M06-2X[S47] and MN15;[S48] the range-separated hybrid functionals ωB97M-V,[S49] ωB97X-D,[S50] CAM-B3LYP[S51] and HSE06[S52] as well as the composite method ωB97-3c;[S53] the local hybrid functionals TMHF,[S54] LHJ-HFcal,[S54] CHYF,[S55] LH20t,[S56] LH23pt,[S57] scLH22t,[S58] scLH22ta,[S58] scLH23t-mBR,[S59] scLH23t-mBR-P[S59] and LH25nP;[S60] the range-separated local hybrid functionals ωLH22t,[S61] ωLH23tdE,[S62] ωLH23tdP,[S62] ωLH23tdB[S62] and ωLH25tdE[S63]

To investigate the effect of long-range EXX admixture in range-separated functionals, we adjusted the β parameter in the LibXC-construction of ωB97X-D, which decomposes the

Coulomb operator as

$$\frac{1}{r_{12}} = \frac{1 - [\alpha + \beta \cdot \text{erf}\,(\omega \cdot r_{12})]}{r_{12}} + \frac{\alpha + \beta \cdot \text{erf}\,(\omega \cdot r_{12})}{r_{12}} \tag{S21}$$

DFT calculations with double hybrid and some other functionals were performed using the ORCA program, version 5.0.2.[S14,S64] All calculations employed the RI approximation with appropriate auxiliary basis sets and a fine integration grid (DefGrid3) as well as improved SCF convergence criteria (VeryTightSCF). Calculations were performed using the def2-QZVPP basis set mentioned above and the DH-SVPD basis set of ref. S65. Calculations were performed with: whe meta-GGA functionals B97M-V,[S66] M06-L[S67] and MN15-L;[S68] the global hybrid functionals B97-1,[S69] M05[S70] and M06;[S47] the range-separated hybrid ωB97X-V;[S71] the double hybrid functionals B2PLYP,[S72] B2GP-PLYP,[S73] mPW2PLYP,[S74] PWPB95,[S75] DSD-BLYP,[S76] DSD-PBEP86,[S77] DSD-PBEB95,[S78] XYG3[S79] and PBE-QIDH;[S80] the range-separated double hybrids ωB2PLYP,[S81] ωPBEPP86,[S82] ωB88PP86,[S82] ωB97X-2,[S83] and ωB97M(2).[S84] We also performed canonical MP2, SCS-MP2 and regularized κ-MP2 calculations using $\kappa = 1.1$.

Fractional-occupation density (FOD) calculations[S85] were also performed in ORCA, using the BHLYP functional,[S46] def2-TZVP basis sets[S23] and an electronic temperature of 15000 K as proposed by Bauer et al..[S86] FODs were visualized using the VMD program.[S87]

Additional Results

Table S1: Contribution of the different correction terms to the relative isomer energies. All values in kcal/mol.

isomer	eq. (S7)	eq. (S8)	eq. (S13)	eq. (S14)	eq. (S12)	eq. (S15)	eq. (S16)
**1**	±0.00	±0.00	±0.00	±0.00	±0.00	±0.00	±0.00
**2**	+0.15	+0.02	+0.07	−0.07	+0.01	−0.06	−0.52
**3**	+0.23	−0.01	+0.03	−0.14	−0.09	−0.11	−0.89
**4**	+0.23	+0.00	+0.03	−0.13	−0.09	−0.14	−0.88
**5**	+0.28	+0.01	−0.14	−0.21	−0.33	−0.33	−1.03
**6**	+0.31	+0.02	+0.07	−0.17	−0.08	−0.19	−1.18
**8**	+0.31	+0.07	+0.08	−0.09	+0.01	−0.04	−1.08
**20**	+0.37	−0.13	−0.73	−0.89	−1.58	−1.80	−2.66
**42**	+0.40	−0.22	−0.53	−0.67	−1.18	−1.32	−2.40
**195**	+0.49	−0.25	−0.79	−1.22	−2.00	−2.69	−3.56
**196**	+0.46	−0.11	−1.02	−0.83	−1.77	−1.59	−2.91
**265**	+0.52	−0.21	−0.89	−1.00	−1.87	−2.33	−2.75
**266**	+0.54	−0.09	−1.27	−1.04	−2.27	−2.43	−3.20
**303**	+0.50	−0.31	−1.02	−1.54	−2.51	−3.32	−3.89
**576**	+0.59	−0.29	−1.38	−1.51	−2.81	−3.29	−3.65
**580**	+0.56	−0.33	−0.65	−0.79	−1.40	−1.63	−2.88
**795**	+0.63	−0.34	−1.77	−1.70	−3.41	−3.52	−4.46
**1748**	+0.79	−0.16	−6.39	−8.24	−14.34	−20.21	−6.72

Table S2: T_1 and D_1 diagnostics of the PNO-LCCSD-F12b/VDZ-F12' wave function and results of an FOD analysis at the BHLYP/def2-TZVP level of theory (at 15000 K) for all investigated C_{60} isomers.[a]

isomer	T_1	D_1	N^{FOD}	n^{FOD}_{mean}	n^{FOD}_{max}	n^{FOD}_{min}
**1**	0.013	0.031	5.146	0.086	0.086	0.086
**2**	0.013	0.036	5.472	0.091	0.133	0.067
**3**	0.013	0.033	5.644	0.094	0.132	0.065
**4**	0.013	0.034	5.643	0.094	0.134	0.065
**5**	0.013	0.042	5.992	0.100	0.172	0.061
**6**	0.013	0.035	5.767	0.096	0.126	0.067
**8**	0.013	0.035	5.848	0.097	0.132	0.068
**20**	0.013	0.043	6.158	0.103	0.172	0.053
**42**	0.013	0.039	6.433	0.107	0.179	0.053
**195**	0.014	0.066	6.453	0.108	0.175	0.064
**196**	0.014	0.055	6.271	0.105	0.212	0.061
**265**	0.013	0.037	6.402	0.107	0.163	0.058
**266**	0.014	0.048	6.721	0.112	0.182	0.046
**303**	0.013	0.040	7.056	0.118	0.183	0.062
**576**	0.014	0.048	6.822	0.114	0.176	0.051
**580**	0.013	0.038	6.326	0.105	0.155	0.064
**795**	0.014	0.065	6.907	0.115	0.189	0.057
**1748**	0.013	0.037	7.776	0.130	0.237	0.087

[a]Values for individual atoms are provided in a separate excel file.

Table S3: Bending angle, maximum atomic contribution to N^{FOD} and relative energies at various coupled-cluster levels for the benzene-based model system discussed in the main text. All energies in kcal/mol.

φ [°]	n_{max}^{FOD}	CCSD	CCSD(T)	CCSDT	CCSDT(Q)	CCSD(T)-cf	CCSDT(Q)-r
90	0.038	0.000	0.000	0.000	0.000	0.000	0.000
95	0.040	0.266	0.257	0.257	0.256	0.256	0.256
100	0.043	1.061	1.027	1.027	1.022	1.022	1.022
105	0.047	2.380	2.304	2.304	2.293	2.294	2.293
110	0.051	4.219	4.083	4.084	4.065	4.066	4.065
115	0.055	6.578	6.365	6.367	6.336	6.337	6.336
120	0.059	9.457	9.150	9.152	9.108	9.110	9.108
125	0.063	12.857	12.439	12.442	12.382	12.385	12.381
130	0.068	16.779	16.235	16.238	16.159	16.164	16.158
135	0.074	21.223	20.537	20.540	20.440	20.447	20.438
140	0.081	26.184	25.344	25.346	25.221	25.234	25.219
145	0.088	31.657	30.650	30.651	30.499	30.518	30.497
150	0.097	37.628	36.446	36.446	36.263	36.292	36.260
155	0.106	44.078	42.717	42.715	42.499	42.540	42.496
160	0.115	50.977	49.439	49.436	49.185	49.239	49.181
165	0.124	58.280	56.578	56.574	56.287	56.358	56.283
170	0.134	65.923	64.085	64.080	63.759	63.848	63.755
175	0.145	73.812	71.884	71.881	71.532	71.638	71.527
180	0.157	81.804	79.863	79.863	79.499	79.617	79.494

Table S4: Relative isomer energies using the expression shown in eq. (S6), identical to eq. 1 in main text, without and with minor modifications. All values in kcal/mol.

isomer	eq. (S6)	eq. (S6)[a]	eq. (S6)[b]	eq. (S6)[c]	eq. (S6)[d]	eq. (S6)[e]
**1**	0.00	0.00	0.00	0.00	0.00	0.00
**2**	37.39	37.37	37.32	37.91	36.90	36.74
**3**	55.93	55.90	55.91	56.82	55.17	55.03
**4**	55.79	55.76	55.74	56.67	55.04	54.89
**5**	68.78	68.76	68.80	69.81	67.47	66.77
**6**	74.60	74.56	74.49	75.78	73.67	73.55
**8**	75.17	75.13	75.11	76.25	74.11	73.74
**20**	92.32	92.30	92.18	94.98	90.73	91.35
**42**	106.46	106.38	106.33	108.85	104.45	104.24
**195**	130.64	130.58	130.02	134.20	128.59	129.51
**196**	131.48	131.39	131.70	134.39	129.71	130.35
**265**	136.12	136.00	135.67	138.87	134.16	134.37
**266**	136.83	136.79	136.78	140.02	134.35	134.36
**303**	136.42	136.27	135.62	140.31	133.47	133.45
**576**	157.95	157.90	157.60	161.60	155.32	155.58
**580**	158.39	158.22	158.11	161.27	156.58	157.04
**795**	170.11	170.05	170.17	174.57	167.33	168.25
**1748**	260.03	259.31	253.96	266.75	256.03	257.31

[a]With the (T*) corrections being replaced by (Ts) corrections). [b]With the PNO-correction (S12) replaced by (S15). [c]Neglecting the post-CCSD(T) correction (S16). [d]Including the correction obtained from n_{max}^{FOD} values using the functional relation in Fig. S2. [e]Replacing the post-CCSD(T) correction (S16) by corrections from n_{max}^{FOD} values using the functional relation in Fig. S3.

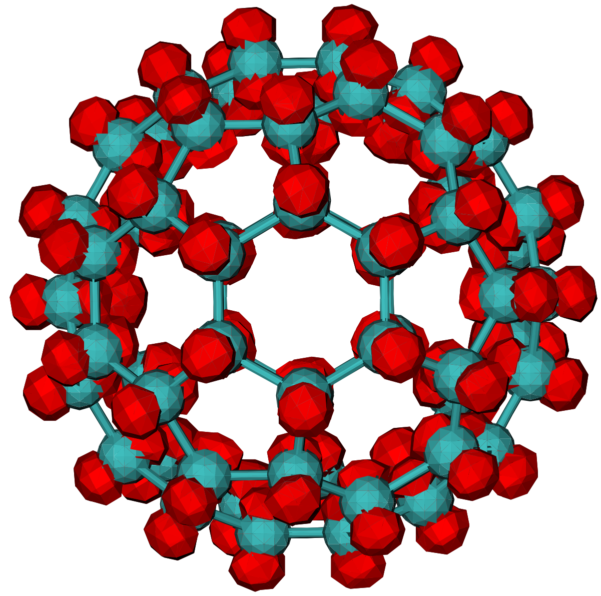

Figure S1: ρ^{FOD} of the I_h symmetric isomer **1** plotted at an isosurface value of 0.005.

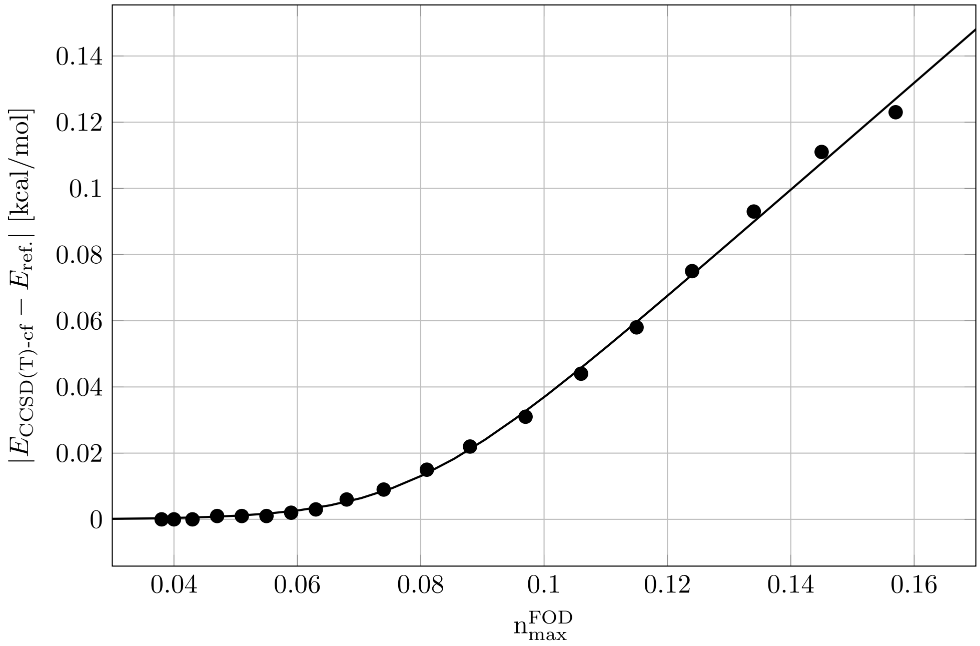

Figure S2: Absolute deviation of the CCSD(T)-cf/DZ results for the benzene-based model system from CCSDT(Q)-r reference energies as a function of n_{max}^{FOD}. A functional relation was fitted to have the form $\frac{A}{B} \cdot \ln\big(1 + \exp(B \cdot (n_{max}^{FOD} - C))\big)$ with A=1.616, B=95.77, C=0.078.

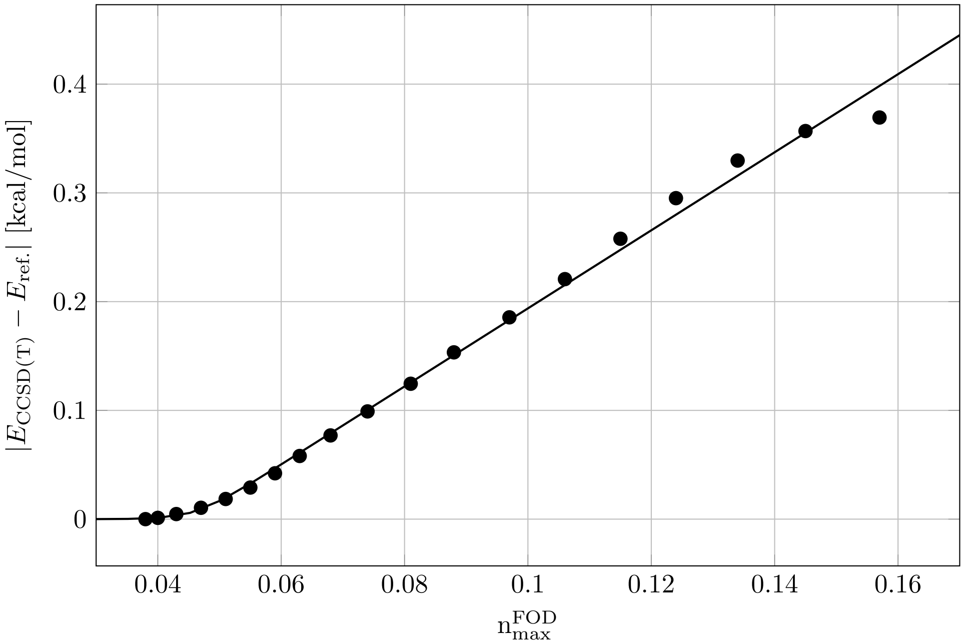

Figure S3: Absolute deviation of the CCSD(T)/DZ results for the benzene-based model system from CCSDT(Q)-r reference energies as a function of n_{max}^{FOD}. A functional relation was fitted to have the form $\frac{A}{B} \cdot \ln\big(1 + \exp(B \cdot (n_{max}^{FOD} - C))\big)$ with A=3.589, B=353.7, C=0.046.

Table S5: Relative isomer energies at the MP2 level using either complete basis-set extrapolation, MP2-F12 calculations with different values of β as described above, or the MP2/CBS* protocol of ref. S1. The mean absolute deviation of the different approaches from the CBS results is also provided. All values in kcal/mol.

		MP2-F12/VDZ-F12'			
isomer	MP2/CBS	($\beta = 0.8$)	($\beta = 0.9$)	($\beta = 1.0$)	MP2/CBS*
**1**	0.00	0.00	0.00	0.00	0.00
**2**	36.74	36.74	36.73	36.76	35.65
**3**	53.75	53.76	53.75	53.74	51.79
**4**	53.79	53.80	53.78	53.79	51.90
**5**	67.19	67.21	67.21	67.22	65.18
**6**	71.42	71.44	71.42	71.44	68.92
**8**	73.96	73.97	73.95	74.02	71.78
**20**	72.94	73.00	73.02	72.88	67.12
**42**	93.08	93.11	93.11	92.90	87.63
**195**	105.19	105.25	105.26	105.02	97.23
**196**	115.08	115.10	115.09	114.98	108.56
**265**	117.13	117.17	117.18	116.96	110.55
**266**	116.21	116.28	116.30	116.21	109.38
**303**	104.08	104.17	104.21	103.91	95.01
**576**	132.80	132.88	132.91	132.62	124.64
**580**	141.86	141.88	141.86	141.53	134.80
**795**	139.45	139.53	139.56	139.23	129.54
**1748**	184.56	184.58	184.58	184.42	164.96
MAD	—	0.10	0.13	0.14	5.72

Table S6: Relative isomer energies of the UPU23 test set[S88] obtained in this and in earlier work. All values in kcal/mol.[a]

isomer	this work	original[S88]
**0a**	5.62	4.82
**0b**	6.28	6.70
**1a**	5.05	4.87
**1b**	3.72	2.97
**1c**	9.40	8.90
**1e**	11.50	11.13
**1f**	13.78	14.41
**1g**	2.57	2.22
**1m**	6.48	5.60
**1p**	2.16	2.02
**2a**	2.79	3.14
**2h**	10.60	10.42
**2p**	0.00	0.00
**3a**	7.18	6.84
**3b**	6.42	6.09
**3d**	5.55	5.42
**4b**	5.41	5.48
**5z**	0.09	0.57
**6p**	3.27	3.32
**7a**	8.16	7.26
**7p**	3.68	3.90
**8d**	6.35	6.43
**9a**	5.29	5.15
**aa**	4.11	3.96

[a]Computed according to eq. S6, equivalent to eq. 1 in main text.

Table S7: Mean absolute deviations (MAD), mean signed deviations (MSD), mean percentage deviations (MPD), and mean absolute (i.e., unsigned) percentage deviations (MAPD) of different DFAs (GGAs, meta-GGAs, global hybrid functionals, range-separated hybrid functionals) from the new reference data for the full C60iso-17 test set and the C60ISO and iso-C60 subsets. All values in kcal/mol.

DFA	MAD	MAD[a]	MAD[b]	MSD	MSD[a]	MSD[b]	MPD	MAPD
BLYP-D4	7.94	6.25	9.63	−7.94	−6.25	−9.63	−6.44	6.44
BP86-D4	7.92	6.35	9.43	−7.92	−6.35	−9.43	−6.40	6.40
PBE-D4	8.04	6.38	9.66	−8.04	−6.38	−9.66	−6.50	6.50
HF-PBE-D4	3.26	2.23	4.33	−0.74	−1.07	0.29	−0.41	2.57
B97-D3	4.57	3.41	5.55	−4.57	−3.41	−5.55	−3.47	3.47
B97M-V	1.78	1.25	2.57	0.82	0.54	1.53	0.91	1.64
M06-L	1.97	1.16	2.79	−0.79	−0.70	−0.50	−0.51	1.58
MN15-L	1.91	1.42	2.44	−0.60	−0.69	−0.19	−0.28	1.64
TPSS-D4	6.22	4.98	7.37	−6.22	−4.98	−7.37	−4.97	4.97
r^2SCAN-3c	1.74	1.09	2.48	0.22	0.08	0.77	0.36	1.51
TPSSh-D4	1.37	0.93	1.80	−0.27	−0.36	0.15	−0.08	1.16
B3LYP-D4	4.65	3.49	6.34	4.65	3.49	6.34	3.88	3.88
O3LYP-D4	1.40	0.98	1.74	−0.48	−0.46	−0.16	−0.23	1.18
B97-1	5.28	3.75	7.46	5.28	3.75	7.46	4.15	4.15
PBE0-D4	7.05	5.29	9.45	7.05	5.29	9.45	5.88	5.88
TPSS0-D4	8.34	6.27	11.08	8.34	6.27	11.08	6.95	6.95
PW6B95-D4	7.14	5.27	9.69	7.14	5.27	9.69	5.81	5.81
BMK	12.19	8.93	16.39	12.19	8.93	16.39	9.63	9.63
BHLYP-D4	22.03	16.70	28.62	22.03	16.70	28.62	17.91	17.91
M05	5.22	3.72	7.34	5.22	3.72	7.34	4.21	4.21
M06	5.22	3.78	7.21	5.22	3.78	7.21	4.24	4.24
M06-2X-D4	16.78	12.53	22.13	16.78	12.53	22.13	13.42	13.42
MN15-D4	9.58	6.85	13.11	9.58	6.85	13.11	7.42	7.42
ωB97M-V	21.80	15.95	28.95	21.80	15.95	28.95	17.20	17.20
ωB97X-D	20.54	15.28	27.14	20.54	15.28	27.14	16.35	16.35
HF-ωB97X-D	22.44	16.80	29.43	22.44	16.80	29.43	18.01	18.01
PBE-ωB97X-D	22.82	17.68	29.36	22.82	17.68	29.36	18.71	18.71
ωB97X-V	24.77	18.51	32.32	24.77	18.51	32.32	19.80	19.80
CAM-B3LYP-D4	19.19	14.43	25.13	19.19	14.43	25.13	15.45	15.45
HSE06-D4	5.20	3.89	7.07	5.20	3.89	7.07	4.40	4.40
ωB97X-3c	25.83	19.40	33.57	25.83	19.40	33.57	20.78	20.78

[a]Restricted to the isomers that are part of the C60ISO subset. [b]Restricted to the isomers that are part of the iso-C60 subset.

Table S8: Mean absolute deviations (MAD), mean signed deviations (MSD), mean percentage deviations (MPD) and mean absolute (i.e., unsigned) percentage deviations (MAPD) of different DFAs (local hybrids, range-separated local hybrids, double hybrids, range separated double hybrids, MP2) from the new reference data for the full C60iso-17 test set and the C60ISO and iso-C60 subsets. All values in kcal/mol.

DFA	MAD	MAD[a]	MAD[b]	MSD	MSD[a]	MSD[b]	MPD	MAPD
TMHF-D4	13.86	10.34	18.35	13.86	10.34	18.35	11.24	11.24
LHJ-HFcal-D4	17.46	13.09	22.85	17.46	13.09	22.85	14.33	14.33
CHYF	5.69	4.15	7.85	5.69	4.15	7.85	4.75	4.75
LH20t-D4	7.99	6.04	10.59	7.99	6.04	10.59	6.72	6.72
LH23pt-D4	9.42	7.07	12.49	9.42	7.07	12.49	7.86	7.86
scLH22t-D4	1.51	1.19	1.71	−1.18	−0.82	−1.36	−0.67	1.22
scLH22ta-D4	2.12	1.65	2.43	−2.02	−1.61	−2.24	−1.45	1.59
scLH23t-mBR-D4	5.79	4.32	7.27	−5.79	−4.32	−7.27	−4.42	4.42
scLH23t-mBR-P-D4	11.19	8.52	14.12	−11.19	−8.52	−14.12	−8.89	8.89
LH25nP-D4	12.04	9.70	14.70	−12.04	−9.70	−14.70	−10.25	10.25
ωLH22t-D4	21.66	16.09	28.51	21.66	16.09	28.51	17.35	17.35
ωLH23tdE-D4	7.10	5.60	9.06	7.10	5.60	9.06	5.87	5.87
ωLH23tdP-D4	1.29	0.90	1.58	−1.04	−0.67	−1.29	−0.80	1.03
ωLH23tdB-D4	9.40	7.18	12.20	9.40	7.18	12.20	7.64	7.64
ωLH25tdE-D4	6.82	5.45	8.64	6.82	5.45	8.64	5.66	5.66
B2PLYP-D4	4.43	2.70	6.50	−3.95	−2.18	−6.00	−2.37	3.20
B2GP-PLYP-D4	3.54	2.13	5.37	−2.33	−0.79	−4.17	−0.89	2.90
mPW2PLYP-D4	1.83	1.58	2.26	−0.08	0.64	−0.85	0.66	1.93
PWPB95-D4	1.61	1.56	1.70	1.42	1.56	1.23	1.59	1.66
DSD-BLYP-D3BJ	5.01	2.88	7.72	−4.14	−1.93	−6.83	−2.22	3.68
DSD-PBEP86-D3BJ	4.62	2.55	7.33	−3.95	−1.84	−6.62	−2.15	3.28
DSD-PBEB95-D3BJ	1.69	1.26	2.40	0.09	0.94	−1.08	0.85	1.61
XYG3	9.28	5.07	14.57	−9.23	−5.04	−14.47	−6.02	6.12
PBE-QIDH	3.47	3.45	3.54	3.47	3.45	3.54	3.71	3.71
mPW2PLYP-D4[c]	2.45	1.52	3.55	−1.49	−0.43	−2.62	−0.52	2.08
PBE-QIDH[c]	2.16	2.39	2.00	2.04	2.34	1.77	2.49	2.55
ωB2PLYP	17.61	13.05	23.27	17.61	13.05	23.27	14.27	14.27
ωB2PLYP[d]	10.78	7.78	14.72	10.78	7.78	14.72	8.73	8.73
ωPBEPP86	3.44	2.33	5.19	3.16	1.98	4.96	2.87	3.11
ωB88PP86	5.86	4.03	8.40	5.86	4.03	8.40	4.95	4.95
ωB97X-2	2.93	1.72	4.81	2.47	1.38	4.14	2.08	2.44
ωB97M(2)	8.06	6.42	10.00	8.06	6.42	10.00	6.93	6.93
HF-D4	44.54	33.74	57.34	44.54	33.74	57.34	35.92	35.92
MP2	18.06	10.71	27.43	−18.06	−10.71	−27.43	−12.41	12.41
SCS-MP2	9.41	4.77	15.80	−9.41	−4.77	−15.80	−6.11	6.11
κ-MP2	23.01	17.41	29.66	23.01	17.41	29.66	18.89	18.89

[a]Restricted to the isomers that are part of the C60ISO subset. [b]Restricted to the isomers that are part of the iso-C60 subset. [c]Using the DH-SVPD basis set of ref. S65. [d]Using a range-separation factor of $\omega = 0.18$.

Table S9: Mean absolute deviations (MAD) and mean signed deviations (MSD) of different DFAs (GGAs, meta-GGAs, global hybrid functionals, range-separated hybrid functionals) from the older reference data of the C60ISO and iso-C60 subsets. All values in kcal/mol.

DFA	MAD[a]	MAD[b]	MSD[a]	MSD[b]
BLYP-D4	10.97	7.14	−10.97	−7.14
BP86-D4	11.06	6.93	−11.06	−6.93
PBE-D4	11.10	7.16	−11.10	−7.16
HF-PBE-D4	6.31	3.71	−5.78	2.79
B97-D3	8.12	3.21	−8.12	−3.06
B97M-V	4.73	4.02	−4.18	4.02
M06-L	5.55	2.33	−5.42	1.99
MN15-L	5.60	2.30	−5.41	2.30
TPSS-D4	9.69	4.88	−9.69	−4.88
r^2SCAN-3c	4.94	3.26	−4.63	3.26
TPSSh-D4	5.18	2.64	−5.07	2.64
B3LYP-D4	2.56	8.84	−1.23	8.84
O3LYP-D4	5.28	2.34	−5.18	2.34
PBE0-D4	2.23	11.95	0.58	11.95
TPSS0-D4	2.58	13.57	1.55	13.57
PW6B95-D4	2.02	12.18	0.56	12.18
BHLYP-D4	11.98	31.12	11.98	31.12
B97-1	2.21	9.95	−0.96	9.95
BMK	4.22	18.88	4.22	18.88
M05	2.27	9.83	−1.00	9.83
M06	2.14	9.70	−0.93	9.70
M06-2X-D4	7.82	24.62	7.82	24.62
MN15-D4	2.14	15.61	2.14	15.61
ωB97M-V	11.23	31.45	11.23	31.45
ωB97X-D	10.56	29.63	10.56	29.63
HF-ωB97X-D	12.09	31.92	12.09	31.92
PBE-ωB97X-D	12.96	31.85	12.96	31.85
ωB97X-V	13.80	34.81	13.80	34.81
CAM-B3LYP-D4	9.71	27.62	9.71	27.62
HSE06-D4	2.54	9.56	−0.82	9.56
ωB97X-3c	14.69	36.07	14.69	36.07

[a]Restricted to the isomers that are part of the C60ISO subset. [b]Restricted to the isomers that are part of the iso-C60 subset.

Table S10: Mean absolute deviations (MAD) and mean signed deviations (MSD) of different DFAs (local hybrids, range-separated local hybrids, double hybrids, range-separated double hybrids, MP2) from the older reference data of the C60ISO and iso-C60 subsets. All values in kcal/mol.

DFA	MAD[a]	MAD[b]	MSD[a]	MSD[b]
TMHF-D4	5.62	20.84	5.62	20.84
LHJ-HFcal-D4	8.38	25.34	8.38	25.34
CHYF	2.52	10.34	−0.56	10.34
LH20t-D4	2.33	13.08	1.32	13.08
LH23pt-D4	2.65	14.99	2.35	14.99
scLH22t-D4	5.58	1.13	−5.54	1.13
scLH22ta-D4	6.32	0.62	−6.32	0.26
scLH23tm-BR-D4	9.03	4.78	−9.03	−4.78
scLH23tm-BR-P-D4	13.24	11.63	−13.24	−11.63
LH25nP-D4	14.41	12.21	−14.41	−12.21
ωLH22t-D4	11.37	31.00	11.37	31.00
ωLH23tdE-D4	1.25	11.56	0.89	11.56
ωLH23tdP-D4	5.38	1.57	−5.38	1.21
ωLH23tdB-D4	2.47	14.69	2.47	14.69
ωLH25tdE-D4	1.20	11.13	0.73	11.13
B2PLYP-D4	7.05	4.53	−6.90	−3.51
B2GP-PLYP-D4	6.14	3.41	−5.50	−1.68
mPW2PLYP-D4	4.95	1.84	−4.07	1.64
PWPB95-D4	3.68	3.72	−3.15	3.72
DSD-BLYP-D3BJ	6.93	5.76	−6.64	−4.34
DSD-PBEP86-D3BJ	6.74	5.37	−6.55	−4.13
DSD-PBEB95-D3BJ	4.46	3.50	−3.78	1.42
XYG3	9.79	12.29	−9.76	−11.97
PBE-QIDH	3.65	6.03	−1.27	6.03
mPW2PLYP-D4[c]	5.46	1.86	−5.15	−0.12
PBE-QIDH[c]	4.12	4.27	−2.38	4.27
ωB2PLYP	8.34	25.76	8.34	25.76
ωB2PLYP[d]	3.15	17.21	3.07	17.21
ωPBEPP86	4.72	7.45	−2.74	7.45
ωB88PP86	3.17	10.90	−0.68	10.90
ωB97X-2	4.62	6.63	−3.34	6.63
ωB97M(2)	2.50	12.49	1.71	12.49
HF-D4	29.02	59.83	29.02	59.83
MP2	15.42	24.94	−15.42	−24.94
SCS-MP2	9.49	13.41	−9.49	−13.31
κ-MP2	12.70	32.15	12.70	32.15

[a]Restricted to the isomers that are part of the C60ISO subset. [b]Restricted to the isomers that are part of the iso-C60 subset. [c]Using the DH-SVPD basis set of ref. S65. [d]Using a range-separation factor of $\omega = 0.18$.

Table S11: Mean absolute deviations (MAD), mean percentage deviations (MPD) and mean absolute (i.e., unsigned) percentage deviations (MAPD) of modified versions of the ωB97X-D functional with different amounts of long-range EXX from the new reference data for the full C60iso-17 set and the C60ISO and iso-C60 subsets. The amount of long-range EXX admixture can be obtained as 0.22 + β (see also eqn (S21)). All values in kcal/mol.

DFA	MAD	MAD[a]	MAD[b]	MPD	MAPD
$\beta = -0.22$	2.05	1.25	2.89	-0.99	1.49
$\beta = +0.00$	3.95	2.75	5.71	3.13	3.13
$\beta = +0.39$	12.46	9.24	16.64	9.95	9.95
$\beta = +0.78$[c]	20.54	15.28	27.14	16.35	16.35

[a]Restricted to the isomers that are part of the C60ISO subset. [b]Restricted to the isomers that are part of the iso-C60 subset. [c]This corresponds to the original formulation of ωB97X-D.

Analysis of strong-correlation contributions in scRSLH functionals by removal of either the $q_{\mathbf{AC}}(\mathbf{r})$ sc-factor or the additional long-range modification. The effect of the strong-correlation correction in range-separated local hybrid functionals was investigated by modifying the energy expression[S62]

$$E_{\mathrm{XC}}^{scRSLH} = E_{\mathrm{X}}^{\mathrm{ex}} + \int 2q_{\mathrm{AC}}(\mathbf{r}) \left[(1 - g(\mathbf{r})) \sum_{\sigma} \left(\Delta e_{\mathrm{SR},\sigma}(\mathbf{r}) + f_{\mathrm{FR}}(\mathbf{r}) \Delta e_{\mathrm{LR},\sigma}(\mathbf{r}) \right) + e_{\mathrm{C}}^{\mathrm{B95}} \right] \mathrm{d}\mathbf{r} \tag{S22}$$

to either

$$E_{\mathrm{XC}}^{scRSLH} = E_{\mathrm{X}}^{\mathrm{ex}} + \int \left[(1 - g(\mathbf{r})) \sum_{\sigma} \left(\Delta e_{\mathrm{SR},\sigma}(\mathbf{r}) + f_{\mathrm{FR}}(\mathbf{r}) \Delta e_{\mathrm{LR},\sigma}(\mathbf{r}) \right) + e_{\mathrm{C}}^{\mathrm{B95}} \right] \mathrm{d}\mathbf{r} \tag{S23}$$

or

$$E_{\mathrm{XC}}^{scRSLH} = E_{\mathrm{X}}^{\mathrm{ex}} + \int 2q_{\mathrm{AC}}(\mathbf{r}) \left[(1 - g(\mathbf{r})) \sum_{\sigma} \left(\Delta e_{\mathrm{SR},\sigma}(\mathbf{r}) \right) + e_{\mathrm{C}}^{\mathrm{B95}} \right] \mathrm{d}\mathbf{r} \tag{S24}$$

In the first instance, the strong-correlation factor in the LMF has been set to 1.0, in the second case the long-range modification term has been removed. Results of the full and the two revised version are compared in Table S12.

Table S12: Mean absolute deviations (MAD), mean percentage deviations (MPD) and mean absolute percentage deviations (MAPD) of modified versions of the ωLH23tE-D4 functional from the new reference data for the full C60iso-17 test set and the C60ISO and iso-C60 subsets. All values in kcal/mol.

DFA	MAD	MAD[a]	MAD[b]	MPD	MAPD
eqn. (S24)	13.55	10.17	17.77	10.90	10.90
eqn. (S23)	18.68	13.96	24.51	15.01	15.01
eqn. (S22)	7.10	5.60	9.06	5.87	5.87
ωLH22t-D4	21.66	16.09	28.51	17.35	17.35

[a]Restricted to the isomers that are part of the C60ISO subset. [b]Restricted to the isomers that are part of the iso-C60 subset.

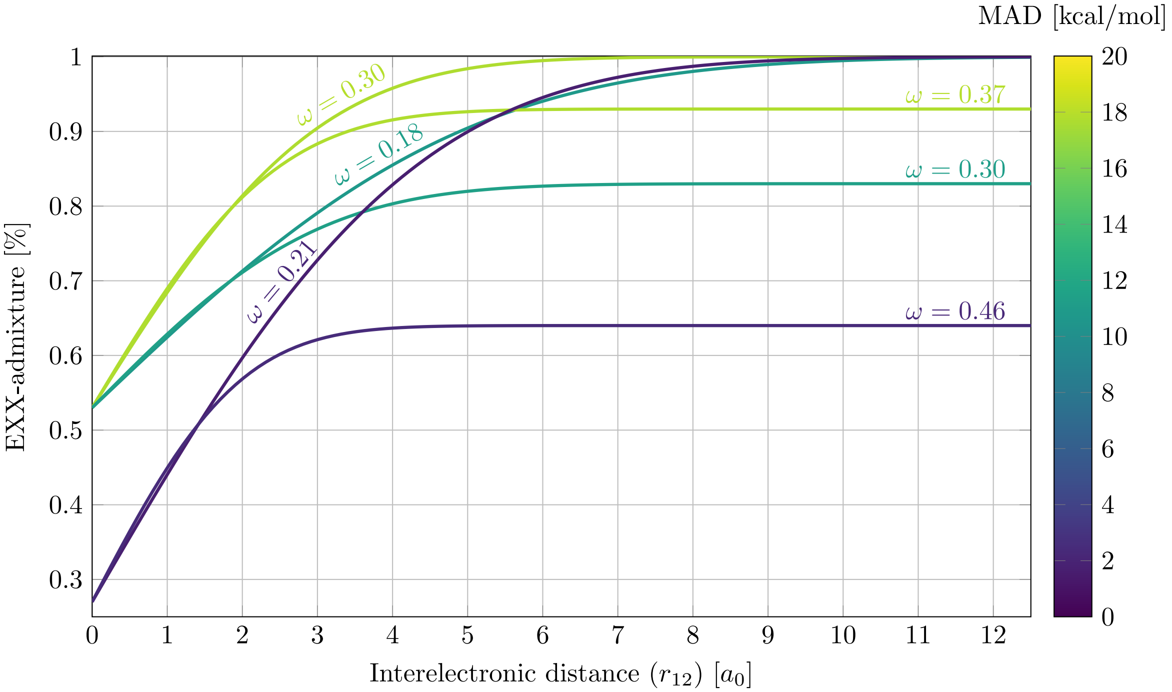

Figure S4: Analysis of the interrelations between performance for C60iso-17 (mean absolute deviation color-coded) and EXX admixture as a function of inter-electronic distance for various modified ωB2PLYP-type double hybrid functionals.

References

(S1) Sure, R.; Hansen, A.; Schwerdtfeger, P.; Grimme, S. Comprehensive Theoretical Study of all 1812 C_60 Isomers. *Phys. Chem. Chem. Phys.* **2017**, *19*, 14296–14305.

(S2) Werner, H.-J.; Knowles, P. J.; Manby, F. R.; Black, J. A.; Doll, K.; Heßelmann, A.; Kats, D.; Köhn, A.; Korona, T.; Kreplin, D. A.; Ma, Q.; Miller, T. F.; Mitrushchenkov, A.; Peterson, K. A.; Polyak, I.; Rauhut, G.; Sibaev, M. The Molpro Quantum Chemistry Package. *J. Chem. Phys.* **2020**, *152*, 144107.

(S3) MOLPRO V2023.2, a Package of *ab initio* Programs, H.-J. Werner, P. J. Knowles, G. Knizia, F. R. Manby, M. Schütz, P. Celani, W. Györffy, D. Kats, T. Korona, R. Lindh, A. Mitrushenkov, G. Rauhut, K. R. Shamasundar, T. B. Adler, R. D. Amos, S. J. Bennie, A. Bernhardsson, A. Berning, D. L. Cooper, M. J. O. Deegan, A. J. Dobbyn, F. Eckert, E. Goll, C. Hampel, A. Hesselmann, G. Hetzer, T. Hrenar, G. Jansen, C. Köppl, S. J. R. Lee, Y. Liu, A. W. Lloyd, Q. Ma, R. A. Mata, A. J. May, S. J. McNicholas, W. Meyer, T. F. Miller III, M. E. Mura, A. Nicklass, D. P. O'Neill, P. Palmieri, D. Peng, K. Pflüger, R. Pitzer, M. Reiher, T. Shiozaki, H. Stoll, A. J. Stone, R. Tarroni, T. Thorsteinsson, M. Wang, and M. Welborn, see `https://www.molpro.net` (accessed 2024-07-22).

(S4) Ma, Q.; Werner, H.-J. Scalable Electron Correlation Methods. 2. Parallel PNO-LMP2-F12 with Near Linear Scaling in the Molecular Size. *J. Chem. Theory Comput.* **2015**, *11*, 5291–5304.

(S5) Ma, Q.; Schwilk, M.; Köppl, C.; Werner, H.-J. Scalable Electron Correlation Methods. 4. Parallel Explicitly Correlated Local Coupled Cluster with Pair Natural Orbitals (PNO-LCCSD-F12). *J. Chem. Theory Comput.* **2017**, *13*, 4871–4896.

(S6) Ma, Q.; Werner, H.-J. Scalable Electron Correlation Methods. 5. Parallel Perturbative

Triples Correction for Explicitly Correlated Local Coupled Cluster with Pair Natural Orbitals. *J. Chem. Theory Comput.* **2018**, *14*, 198–215.

(S7) Peterson, K. A.; Adler, T. B.; Werner, H.-J. Systematically Convergent Basis Sets for Explicitly Correlated Wavefunctions: The Atoms H, He, B–Ne, and Al–Ar. *J. Chem. Phys.* **2008**, *128*, 084102.

(S8) Adler, T. B.; Knizia, G.; Werner, H.-J. A Simple and Efficient CCSD(T)-F12 Approximation. *J. Chem. Phys.* **2007**, *127*, 221106.

(S9) Peterson, K. A.; Kesharwani, M. K.; Martin, J. M. L. The cc-pV5Z-F12 basis set: reaching the basis set limit in explicitly correlated calculations. *Mol. Phys.* **2015**, *113*, 1551–1558.

(S10) Knizia, G.; Adler, T. B.; Werner, H.-J. Simplified CCSD(T)-F12 Methods: Theory and Benchmarks. *J. Chem. Phys.* **2009**, *130*, 054104.

(S11) Marchetti, O.; Werner, H.-J. Accurate Calculations of Intermolecular Interaction Energies Using Explicitly Correlated Coupled Cluster Wave Functions and a Dispersion-Weighted MP2 Method. *J. Phys. Chem. A* **2009**, *113*, 11580–11585.

(S12) Riplinger, C.; Sandhoefer, B.; Hansen, A.; Neese, F. Natural Triple Excitations in Local Coupled Cluster Calculations with Pair Natural Orbitals. *J. Chem. Phys.* **2013**, *139*, 134101.

(S13) Goodson, D. Z. Extrapolating the Coupled-Cluster Sequence toward the Full Configuration-Interaction Limit. *J. Chem. Phys.* **2002**, *116*, 6948–6956.

(S14) Neese, F. Software update: The ORCA program system—Version 5.0. *WIREs Comp. Mol. Sci.* **2022**, *12*, e1606.

(S15) Dunning, T. H. Gaussian Basis Sets for Use in Correlated Molecular Calculations. I. The Atoms Boron through Neon and Hydrogen. *J. Chem. Phys.* **1989**, *90*, 1007–1023.

(S16) Weigend, F.; Köhn, A.; Hättig, C. Efficient Use of the Correlation Consistent Basis Sets in Resolution of the Identity MP2 Calculations. *J. Chem. Phys.* **2002**, *116*, 3175–3183.

(S17) Weigend, F. A Fully Direct RI-HF Algorithm: Implementation, Optimised Auxiliary Basis Sets, Demonstration of Accuracy and Efficiency. *Phys. Chem. Chem. Phys.* **2002**, *4*, 4285–4291.

(S18) Feller, D. Application of systematic sequences of wave functions to the water dimer. *J. Chem. Phys.* **1992**, *96*, 6104–6114.

(S19) MRCC, a Quantum Chemical Program Suite written by M. Kállay, P. R. Nagy, D. Mester, Z. Rolik, G. Samu, J. Csontos, J. Csóka, P. B. Szabó, L. Gyevi-Nagy, B. Hégely, I. Ladjánszki, L. Szegedy, B. Ladóczki, K. Petrov, M. Farkas, P. Mezei and Á. Ganyecz. See `www.mrcc.hu` (accessed 2022-10-27).

(S20) Kállay, M.; Nagy, P. R.; Mester, D.; Rolik, Z.; Samu, G.; Csontos, J.; Csóka, J.; Szabó, P. B.; Gyevi-Nagy, L.; Hégely, B.; Ladjánszki, I.; Szegedy, L.; Ladóczki, B.; Petrov, K.; Farkas, M.; Mezei, P. D.; Ganyecz, Á. The MRCC program system: Accurate quantum chemistry from water to proteins. *J. Chem. Phys.* **2020**, *152*, 074107.

(S21) TURBOMOLE V7.9 2025, a Development of University of Kalsruhe and Forschungszentrum Karlsruhe GmbH, 1989-2007, TURBOMOLE GmbH, since 2007; Available from
`http://www.turbomole.com` (accessed 2026-03-05).

(S22) Franzke, Y. J.; Holzer, C.; Andersen, J. H.; Begušić, T.; Bruder, F.; Coriani, S.; Della Sala, F.; Fabiano, E.; Fedotov, D. A.; Fürst, S.; Gillhuber, S.; Grotjahn, R.; Kaupp, M.; Kehry, M.; Krstić, M.; Mack, F.; Majumdar, S.; Nguyen, B. D.; Parker, S. M.; Pauly, F.; Pausch, A.; Perlt, E.; Phun, G. S.; Rajabi, A.; Rappoport, D.; Samal, B.; Schrader, T.; Sharma, M.; Tapavicza, E.; Treß, R. S.; Voora, V.;

Wodyński, A.; Yu, J. M.; Zerulla, B.; Furche, F.; Hättig, C.; Sierka, M.; Tew, D. P.; Weigend, F. TURBOMOLE: Today and Tomorrow. *J. Chem. Theory Comput.* **2023**, *19*, 6859–6890.

(S23) Weigend, F.; Ahlrichs, R. Balanced Basis Sets of Split Valence, Triple Zeta Valence and Quadruple Zeta Valence Quality for H to Rn: Design and Assessment of Accuracy. *Phys. Chem. Chem. Phys.* **2005**, *7*, 3297–3305.

(S24) Sierka, M.; Hogekamp, A.; Ahlrichs, R. Fast Evaluation of the Coulomb Potential for Electron Densities Using Multipole Accelerated Resolution of Identity Approximation. *J. Chem. Phys.* **2003**, *118*, 9136–9148.

(S25) Holzer, C. An Improved Seminumerical Coulomb and Exchange Algorithm for Properties and Excited States in Modern Density Functional Theory. *J. Chem. Phys.* **2020**, *153*, 184115.

(S26) Caldeweyher, E.; Ehlert, S.; Hansen, A.; Neugebauer, H.; Spicher, S.; Bannwarth, C.; Grimme, S. A Generally Applicable Atomic-Charge Dependent London Dispersion Correction. *J. Chem. Phys.* **2019**, *150*, 154122.

(S27) Becke, A. D. Density-Functional Exchange-Energy Approximation with Correct Asymptotic Behavior. *Phys. Rev. A* **1988**, *38*, 3098–3100.

(S28) Lee, C.; Yang, W.; Parr, R. G. Development of the Colle-Salvetti Correlation-Energy Formula into a Functional of the Electron Density. *Phys. Rev. B* **1988**, *37*, 785–789.

(S29) Miehlich, B.; Savin, A.; Stoll, H.; Preuss, H. Results Obtained with the Correlation Energy Density Functionals of Becke and Lee, Yang and Parr. *Chem. Phys. Lett.* **1989**, *157*, 200–206.

(S30) Perdew, J. P. Density-Functional Approximation for the Correlation Energy of the Inhomogeneous Electron Gas. *Phys. Rev. B* **1986**, *33*, 8822–8824.

(S31) Perdew, J. P. Erratum: Density-Functional Approximation for the Correlation Energy of the Inhomogeneous Electron Gas. *Phys. Rev. B* **1986**, *34*, 7406–7406.

(S32) Perdew, J. P.; Burke, K.; Ernzerhof, M. Generalized Gradient Approximation Made Simple. *Phys. Rev. Lett.* **1996**, *77*, 3865–3868.

(S33) Perdew, J. P.; Burke, K.; Ernzerhof, M. Generalized Gradient Approximation Made Simple [Phys. Rev. Lett. 77, 3865 (1996)]. *Phys. Rev. Lett.* **1997**, *78*, 1396–1396.

(S34) Grimme, S. Semiempirical GGA-Type Density Functional Constructed with a Long-Range Dispersion Correction. *J. Comput. Chem.* **2006**, *27*, 1787–1799.

(S35) Grimme, S.; Ehrlich, S.; Goerigk, L. Effect of the Damping Function in Dispersion Corrected Density Functional Theory. *J. Comput. Chem.* **2011**, *32*, 1456–1465.

(S36) Tao, J.; Perdew, J. P.; Staroverov, V. N.; Scuseria, G. E. Climbing the Density Functional Ladder: Nonempirical Meta–Generalized Gradient Approximation Designed for Molecules and Solids. *Phys. Rev. Lett.* **2003**, *91*, 146401.

(S37) Grimme, S.; Hansen, A.; Ehlert, S.; Mewes, J.-M. r2SCAN-3c: A "Swiss Army Knife" Composite Electronic-Structure Method. *J. Chem. Phys.* **2021**, *154*, 064103.

(S38) Staroverov, V. N.; Scuseria, G. E.; Tao, J.; Perdew, J. P. Comparative Assessment of a New Nonempirical Density Functional: Molecules and Hydrogen-Bonded Complexes. *J. Chem. Phys.* **2003**, *119*, 12129–12137.

(S39) Staroverov, V. N.; Scuseria, G. E.; Tao, J.; Perdew, J. P. Erratum: "Comparative Assessment of a New nonempirical Density Functional: Molecules and Hydrogen-Bonded Complexes"[J. Chem. Phys. 119, 12129 (2003)]. *J. Chem. Phys.* **2004**, *121*, 11507–11507.

(S40) Becke, A. D. Density-Functional Thermochemistry. III. The Role of Exact Exchange. *J. Chem. Phys.* **1993**, *98*, 5648–5652.

(S41) Cohen, A. J.; Handy, N. C. Dynamic Correlation. *Mol. Phys.* **2001**, *99*, 607–615.

(S42) Adamo, C.; Barone, V. Toward Reliable Density Functional Methods without Adjustable Parameters: The PBE0 Model. *J. Chem. Phys.* **1999**, *110*, 6158–6170.

(S43) Grimme, S. Accurate Calculation of the Heats of Formation for Large Main Group Compounds with Spin-Component Scaled MP2 Methods. *J. Phys. Chem. A* **2005**, *109*, 3067–3077.

(S44) Zhao, Y.; Truhlar, D. G. Design of Density Functionals That Are Broadly Accurate for Thermochemistry, Thermochemical Kinetics, and Nonbonded Interactions. *J. Phys. Chem. A* **2005**, *109*, 5656–5667.

(S45) Boese, A. D.; Martin, J. M. L. Development of Density Functionals for Thermochemical Kinetics. *J. Chem. Phys.* **2004**, *121*, 3405–3416.

(S46) Holthausen, M. C.; Heinemann, C.; Cornehl, H. H.; Koch, W.; Schwarz, H. The Performance of Density-Functional/Hartree-Fock Hybrid Methods: Cationic Transition-Metal Methyl Complexes MCH_3^+ (M=Sc–Cu,La,Hf–Au). *J. Chem. Phys.* **1995**, *102*, 4931–4941.

(S47) Zhao, Y.; Truhlar, D. G. The M06 Suite of Density Functionals for Main Group Thermochemistry, Thermochemical Kinetics, Noncovalent Interactions, Excited States, and Transition Elements: Two New Functionals and Systematic Testing of Four M06-Class Functionals and 12 Other Functionals. *Theor. Chem. Acc.* **2008**, *120*, 215–241.

(S48) Yu, H. S.; He, X.; Li, S. L.; Truhlar, D. G. MN15: A Kohn–Sham Global-Hybrid Exchange-Correlation Density Functional with Broad Accuracy for Multi-Reference and Single-Reference Systems and Noncovalent Interactions. *Chem. Sci.* **2016**, *7*, 5032–5051.

(S49) Mardirossian, N.; Head-Gordon, M. ωB97M-V: A Combinatorially Optimized, Range-Separated Hybrid, Meta-GGA Density Functional with VV10 Nonlocal Correlation. *J. Chem. Phys.* **2016**, *144*, 214110.

(S50) Chai, J.-D.; Head-Gordon, M. Long-Range Corrected Hybrid Density Functionals with Damped Atom–Atom Dispersion Corrections. *Phys. Chem. Chem. Phys.* **2008**, *10*, 6615–6620.

(S51) Yanai, T.; Tew, D. P.; Handy, N. C. A New Hybrid Exchange-Correlation Functional Using the Coulomb-Attenuating Method (CAM-B3LYP). *Chem. Phys. Lett.* **2004**, *393*, 51–57.

(S52) Krukau, A. V.; Vydrov, O. A.; Izmaylov, A. F.; Scuseria, G. E. Influence of the Exchange Screening Parameter on the Performance of Screened Hybrid Functionals. *J. Chem. Phys.* **2006**, *125*, 224106.

(S53) Müller, M.; Hansen, A.; Grimme, S. ωB97X-3c: A Composite Range-Separated Hybrid DFT Method with a Molecule-Optimized Polarized Valence Double-ζ Basis Set. *J. Chem. Phys.* **2023**, *158*, 014103.

(S54) Holzer, C.; Franzke, Y. J. A Local Hybrid Exchange Functional Approximation from First Principles. *J. Chem. Phys.* **2022**, *157*, 034108.

(S55) Holzer, C.; Franzke, Y. J. A General and Transferable Local Hybrid Functional for Electronic Structure Theory and Many-Fermion Approaches. *J. Chem. Theory Comput.* **2025**, *21*, 202–217.

(S56) Haasler, M.; Maier, T. M.; Grotjahn, R.; Gückel, S.; Arbuznikov, A. V.; Kaupp, M. A Local Hybrid Functional with Wide Applicability and Good Balance between (De)Localization and Left–Right Correlation. *J. Chem. Theory Comput.* **2020**, *16*, 5645–5657.

(S57) Haasler, M.; Maier, T. M.; Kaupp, M. Toward a correct treatment of core properties with local hybrid functionals. *J. Comput. Chem.* **2023**, *44*, 2461–2477.

(S58) Wodyński, A.; Kaupp, M. Local Hybrid Functional Applicable to Weakly and Strongly Correlated Systems. *J. Chem. Theory Comput.* **2022**, *18*, 6111–6123.

(S59) Wodyński, A.; Arbuznikov, A. V.; Kaupp, M. Strong-correlation density functionals made simple. *J. Chem. Phys.* **2023**, *158*, 244117.

(S60) Wodyński, A.; Kaupp, M. Local-Hybrid Functional With a Composite Local Mixing Function Built From a Neural Network and a Strong-Correlation Model. *J. Comput. Chem.* **2026**, *47*, e70294.

(S61) Fürst, S.; Haasler, M.; Grotjahn, R.; Kaupp, M. Full Implementation, Optimization, and Evaluation of a Range-Separated Local Hybrid Functional with Wide Accuracy for Ground and Excited States. *J. Chem. Theory Comput.* **2023**, *19*, 488–502.

(S62) Fürst, S.; Kaupp, M.; Wodyński, A. Range-Separated Local Hybrid Functionals with Small Fractional-Charge and Fractional-Spin Errors: Escaping the Zero-Sum Game of DFT Functionals. *J. Chem. Theory Comput.* **2023**, *19*, 8639–8653.

(S63) Wodyński, A.; Kaupp, M. ́Beyond-Zero-Sum ́Range-Separated Local Hybrid Functional with Improved Dynamical Correlation. *J. Chem. Theory Comput.* **2025**, *21*, 7419–7429.

(S64) Neese, F. The ORCA Program System. *WIREs Comp. Mol. Sci.* **2012**, *2*, 73–78.

(S65) Brémond, É.; Ciofini, I.; Sancho-García, J. C.; Adamo, C. Double-Hybrid Functionals and Tailored Basis Set: Fullerene (C60) Dimer and Isomers as Test Cases. *J. Phys. Chem. A* **2019**, *123*, 10040–10046.

(S66) Mardirossian, N.; Head-Gordon, M. Mapping the Genome of Meta-Generalized Gradient Approximation Density Functionals: The Search for B97M-V. *J. Chem. Phys.* **2015**, *142*, 074111.

(S67) Zhao, Y.; Truhlar, D. G. A New Local Density Functional for Main-Group Thermochemistry, Transition Metal Bonding, Thermochemical Kinetics, and Noncovalent Interactions. *J. Chem. Phys.* **2006**, *125*, 194101.

(S68) Yu, H. S.; He, X.; Truhlar, D. G. MN15-L: A New Local Exchange-Correlation Functional for Kohn–Sham Density Functional Theory with Broad Accuracy for Atoms, Molecules, and Solids. *J. Chem. Theory Comput.* **2016**, *12*, 1280–1293.

(S69) Hamprecht, F. A.; Cohen, A. J.; Tozer, D. J.; Handy, N. C. Development and Assessment of New Exchange-Correlation Functionals. *J. Chem. Phys.* **1998**, *109*, 6264–6271.

(S70) Zhao, Y.; Schultz, N. E.; Truhlar, D. G. Exchange-Correlation Functional with Broad Accuracy for Metallic and Nonmetallic Compounds, Kinetics, and Noncovalent Interactions. *J. Chem. Phys.* **2005**, *123*, 161103.

(S71) Mardirossian, N.; Head-Gordon, M. ωB97X-V: A 10-Parameter, Range-Separated Hybrid, Generalized Gradient Approximation Density Functional with Nonlocal Correlation, Designed by a Survival-of-the-Fittest Strategy. *Phys. Chem. Chem. Phys.* **2014**, *16*, 9904–9924.

(S72) Grimme, S. Semiempirical Hybrid Density Functional with Perturbative Second-Order Correlation. *J. Chem. Phys.* **2006**, *124*, 034108.

(S73) Karton, A.; Tarnopolsky, A.; Lamère, J.-F.; Schatz, G. C.; Martin, J. M. L. Highly Accurate First-Principles Benchmark Data Sets for the Parametrization and Validation of Density Functional and Other Approximate Methods. Derivation of a Robust,

Generally Applicable, Double-Hybrid Functional for Thermochemistry and Thermochemical Kinetics. *J. Phys. Chem. A* **2008**, *112*, 12868–12886.

(S74) Schwabe, T.; Grimme, S. Towards Chemical Accuracy for the Thermodynamics of Large Molecules: New Hybrid Density Functionals Including Non-Local Correlation Effects. *Phys. Chem. Chem. Phys.* **2006**, *8*, 4398–4401.

(S75) Goerigk, L.; Grimme, S. Efficient and Accurate Double-Hybrid-Meta-GGA Density Functionals—Evaluation with the Extended GMTKN30 Database for General Main Group Thermochemistry, Kinetics, and Noncovalent Interactions. *J. Chem. Theory Comput.* **2011**, *7*, 291–309.

(S76) Kozuch, S.; Gruzman, D.; Martin, J. M. L. DSD-BLYP: A General Purpose Double Hybrid Density Functional Including Spin Component Scaling and Dispersion Correction. *J. Phys. Chem. C* **2010**, *114*, 20801–20808.

(S77) Kozuch, S.; Martin, J. M. L. DSD-PBEP86: In Search of the Best Double-Hybrid DFT with Spin-Component Scaled MP2 and Dispersion Corrections. *Phys. Chem. Chem. Phys.* **2011**, *13*, 20104–20107.

(S78) Kozuch, S.; Martin, J. M. L. Spin-Component-Scaled Double Hybrids: An Extensive Search for the Best Fifth-Rung Functionals Blending DFT and Perturbation Theory. *J. Comput. Chem.* **2013**, *34*, 2327–2344.

(S79) Zhang, Y.; Xu, X.; Goddard, W. A. Doubly Hybrid Density Functional for Accurate Descriptions of Nonbond Interactions, Thermochemistry, and Thermochemical Kinetics. *Proc. Natl. Acad. Sci.* **2009**, *106*, 4963–4968.

(S80) Brémond, É.; Sancho-García, J. C.; Pérez-Jiménez, Á. J.; Adamo, C. Communication: Double-Hybrid Functionals from Adiabatic-Connection: The QIDH Model. *J. Chem. Phys.* **2014**, *141*, 031101.

(S81) Casanova-Páez, M.; Dardis, M. B.; Goerigk, L. ωB2PLYP and ωB2GPPLYP: The First Two Double-Hybrid Density Functionals with Long-Range Correction Optimized for Excitation Energies. *J. Chem. Theory Comput.* **2019**, *15*, 4735–4744.

(S82) Casanova-Páez, M.; Goerigk, L. Time-Dependent Long-Range-Corrected Double-Hybrid Density Functionals with Spin-Component and Spin-Opposite Scaling: A Comprehensive Analysis of Singlet–Singlet and Singlet–Triplet Excitation Energies. *J. Chem. Theory Comput.* **2021**, *17*, 5165–5186.

(S83) Chai, J.-D.; Head-Gordon, M. Long-range corrected double-hybrid density functionals. *J. Chem. Phys.* **2009**, *131*, 174105.

(S84) Mardirossian, N.; Head-Gordon, M. Survival of the Most Transferable at the Top of Jacob's Ladder: Defining and Testing the ωB97M(2) Double Hybrid Density Functional. *J. Chem. Phys.* **2018**, *148*, 241736.

(S85) Grimme, S.; Hansen, A. A Practicable Real-Space Measure and Visualization of Static Electron-Correlation Effects. *Angew. Chem., Int. Ed.* **2015**, *54*, 12308–12313.

(S86) Bauer, C. A.; Hansen, A.; Grimme, S. The Fractional Occupation Number Weighted Density as a Versatile Analysis Tool for Molecules with a Complicated Electronic Structure. *Chem. Eur. J.* **2017**, *23*, 6150–6164.

(S87) Humphrey, W.; Dalke, A.; Schulten, K. VMD: Visual Molecular Dynamics. *Journal of Molecular Graphics* **1996**, *14*, 33–38.

(S88) Kruse, H.; Mladek, A.; Gkionis, K.; Hansen, A.; Grimme, S.; Sponer, J. Quantum Chemical Benchmark Study on 46 RNA Backbone Families Using a Dinucleotide Unit. *J. Chem. Theory Comput.* **2015**, *11*, 4972–4991.
